# Association Between Cognitive Function and Emotion, Sleep, Frailty, and Nutrition in Hospitalized Patients

**DOI:** 10.1002/brb3.70170

**Published:** 2024-11-28

**Authors:** Nan Wang, Qunying Zhang, Peng Li, Lilan Guo, Xiaoman Wu, Qiuyun Tu

**Affiliations:** ^1^ Department of Gerontology and Geriatrics Fifth Affiliated Hospital of Sun Yat‐sen University Zhuhai Guangdong China

**Keywords:** cognitive function, emotion, frailty, nutrition, sleep quality

## Abstract

**Background:**

With the rapid increase in China's aging population, cognitive impairment in the elderly has become a significant public health issue.

**Aims:**

In this study we performed a cross‐sectional analysis to comprehensively investigate the relationship between cognitive function and emotion, sleep, frailty, nutrition, and clinical variables in hospitalized geriatric patients according to age group and sex. We determined the most important risk factors for cognitive impairment.

**Method:**

A total of 1121 inpatients were recruited from the Department of Gerontology at the Fifth Affiliated Hospital of Sun Yat‐sen University, China, from August 2023 to April 2024. Cognitive assessment was performed using the Mini‐Mental State Examination scale and Montreal Cognitive Assessment. The sleep quality was evaluated based on the Pittsburgh Sleep Quality Index, and anxiety and depression were evaluated based on the Hamilton Anxiety Scale and Hamilton Depression Scale.

**Results:**

Sex and age differences existed with respect to cognition, emotion, and sleep quality. After full adjustment, age, education level, working status, hemoglobin level, activities of daily living, Hamilton Depression Scale, and Pittsburgh Sleep Quality Index scores were significantly and independently associated with cognitive impairment.

**Discussion:**

Geriatric patients with a better mood, sleep and nutrition status, higher education level, and more social engagement performance had superior cognitive function. Interventions, such as valuing education, improving sleep, relaxing emotions, preventing anemia, and adjusting lifestyle, may help prevent the development of cognitive deficits. Elderly and female patients required special attention.

**Conclusions:**

Various factors were shown to contribute to maintenance of cognitive function.

## Graphical abstract

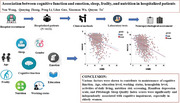

## 1 Introduction

With the rapid growth of China's aging population, cognitive impairment in the elderly has become an important public health problem. China has the highest number of dementia patients worldwide. Moreover, dementia brings a heavy economic and social burden to the public health system (Zhang et al. 2019). Mild cognitive impairment refers to a clinical stage between normal aging and dementia. It is essential to identify this group not only to develop interventions to reduce individual suffering but also because this represents a group at increased risk of developing dementia. Indeed, the early prediction, identification, and prevention of dementia are extremely important. Previous studies have suggested that individuals who are elderly, women, or living in rural areas had higher prevalence of mild cognitive impairment (Xue et al. 2018). It is necessary to strengthen monitoring of elderly Chinese residents with these characteristics to identify and intervene before the onset of dementia. However, information facilitating the early identification of dementia risk factors and prevention among geriatric patients is incomplete. Further relevant studies are recommended in China.

It has been previously documented that the elderly were prone to experience cognitive impairment, sleep disorders, depression, anxiety, frailty, and malnutrition, all of which have a great impact on quality of life (Han et al. 2021; Bartrés‐Faz et al. 2023; Ma et al. 2020). The prevalence of cognitive impairment is high in the aging population, affects mood and sleep, and increases the risk of elderly chronic diseases. Also, depression, anxiety, sleep disorders, and frailty exacerbate cognitive decline (Ward et al. 2022; Barrera‐Caballero et al. 2021; Cladder‐Micus et al. 2018). Considering these strong associations with cognitive impairment in the elderly based on previous studies, future corollary studies should investigate the confounding factors, for example, depression, sleep disturbances, and cognitive impairment in the elderly are likely to be interrelated. However, limited empirical research has analyzed the complex associations between these factors and cognitive function. Whether depression, sleep disturbances, and cognitive impairment among the elderly are independently related or influenced by other factors has not been established. In addition, females are more likely to have cognitive impairment and affective disorders with higher fatigue and sleep disturbances than males (Götze et al. 2020; Niu et al. 2023; Faravelli et al. 2013). These findings suggest that age and sex are important specific factors to consider when evaluating the correlation between cognitive function and related factors.

Furthermore, some studies have reported downwards trend of cognitive impairment, may resulting from modifiable factors management, which were defined as medical elements that are intervenable before dementia occurs (Wolters et al. 2020). Vascular risk factors including blood pressure, plasma glucose, and body mass index (BMI) due to smoking accelerate the progression of neurodegenerative pathology (Wu et al. 2016; Satizabal et al. 2016; Tariq and Barber 2018). Chronic illness, anemia, and physical activity were found for associations of dementia and cognitive impairment in midlife to late life (Zhang et al. 2022). These modifiable risk factors should be identified and considered in dementia prevention.

To address the issue, we conducted a cross‐sectional analysis to determine the relationship between cognitive function, sleep, mood, frailty, nutrition, illness, and medication history in hospitalized patients. In addition, we collected and analyzed important epidemiologic, demographic, clinical, and laboratory factors that may influence cognitive function. We analyzed cognitive function and multiple risk factors from a comprehensive perspective according to age and sex. We focused on evaluating the associations and then identified the factors with high‐level significance. The aim of this study was to identify important risk factors of cognitive impairment to design appropriate and measured strategies for the prevention of dementia and promote healthy aging.

## 2 Materials and Methods

### 2.1 Study Design and Participants

Participants for this cross‐sectional study were enrolled from the Department of Gerontology of Fifth Affiliated Hospital of Sun Yat‐sen University from August 2023 to April 2024. The inclusion criteria included the following: (1) age ≥ 45 years and (2) adequate audiovisual abilities to complete the necessary assessments. The exclusion criteria included the following: (1) failure to provide general demographic information, (2) blindness or speech impairment, and (3) serious neurologic or psychiatric disorders. This study was approved by the Institutional Ethics Committee of Fifth Hospital of Sun Yat‐sen University.

### 2.2 Clinical Data and Blood Tests

All subjects completed a detailed questionnaire at the time of enrollment. Basic data, sociodemographic, and medical information were collected, including age, gender, height, weight, working status, education level, smoking history, and illness and medication histories. Physical examination, including height and weight, were measured with the participants wearing underwear but no shoes. BMI was calculated by weight in kilograms divided by the square of the height in meters. Blood pressure was measured after 10 min of rest, and the average value was calculated. Venous blood was collected 10 h after fasting, and some blood samples were used to assess biochemical parameters, including routine hematologic tests and the serum creatinine, alanine aminotransferase, fasting blood glucose, serum total protein (TP), serum albumin, and serum lipid levels.

### 2.3 Cognitive Function, Emotion, Sleep, Frailty, and Nutrition

The Chinese versions of the Mini‐Mental State Examination (MMSE) and Montreal Cognitive Assessment (MoCA) were used to evaluate cognitive function (Katzman et al. 1988; Lu et al. 2011; Huan and Heyong 2024). MMSE covers attention and computation, orientation to place and time, immediate memory, delayed memory, language, and visual space, with a total score on the scale between 0 and 30. The test result is closely related to the literacy level as follows: illiteracy > 17 points, primary > 20 points, and junior high school and above > 24 points. The MoCA has sensitivity for detecting mild cognitive impairment and is a fast assessment tool for cognitive deficit detection. The MoCA encompasses memory, language, abstract thinking, attention and concentration, computing power, visual skills, executive function, and orientation. The total score is 30 points; a score > 26 points is considered normal. If the average years of education are < 12 years, 1 point is added. Depression was assessed using the Hamilton Depression Scale (HAMD), and anxiety was assessed using the Hamilton Anxiety Scale (HAMA). The sleep quality was evaluated using the Pittsburgh Sleep Quality Index (PSQI). Frailty was evaluated using the Frail scale, and nutrition risk was evaluated using the Nutrition Risk Screening (NRS)‐2002. The evaluation of activities of daily living (ADL) was performed with the Barthel Index.

### 2.4 Statistical Analysis

The Kolmogorov–Smirnov test was used for normality analysis. Data with a normal distribution are expressed as the mean ± SD with differences determined using independent‐sample *t* tests. Data with a non‐normal distribution are expressed as the median and interquartile range. Differences in variables among groups were analyzed using the Mann–Whitney *U* test. Categorical data are shown as numbers and differences in variables among groups and were analyzed using a *χ* ^2^ test or Fisher probabilities. Bonferroni correction was used to deal with multiplicity. The Spearman's rank correlation test was used to assess the correlation between two variables. Multiple regression analyses were used to adjust for possible confounding variables. A *p *< 0.05 was considered to indicate statistical significance. All data were analyzed using SPSS 19.0 statistical software.

## 3 Results

### 3.1 Basic Data and Clinical Variables of Participants Based on Age and Sex

Table 1 shows the basic and clinical characteristics according to age and sex. All variables, except for BMI, high‐density lipoprotein cholesterol, alanine aminotransferase, total bilirubin, and TP, were significantly different between the two age groups (*p *< 0.05). The PSQI, HAMD, HAMA, NRS‐2002, and Frailty scores increased significantly with age (*p *< 0.01). In contrast, the MMSE and MoCA total scores decreased with age (*p* < 0.01). There were sex differences for all variables, except the NRS‐2002 and Frailty scores, in the same age group. Women had a significantly lower level of education, working status, hemoglobin (HGB) level, and MMSE and MoCA scores and higher PSQI, HAMD, and HAMA scores than men (all *p* < 0.05).

**TABLE 1:** Basic characteristics of the subjects based on age groups and gender.

	< 60 years				> 60 years			
	Total (*n* = 433)	Male (*n* = 153)	Female (*n* = 280)	*p* value	Total (*n* = 688)	Male (*n* = 309)	Female (*n* = 379)	*p* value
Age (years)	53.99 ± 5.19	54.14 ± 5.56	53.91 ± 4.98	0.679	71.40 ± 7.62^**^	71.42 ± 7.82^**^	71.37 ± 7.48^**^	< 0.01
BMI (kg/m^2^)	24.11 ± 3.41	24.99 ± 3.01	23.64 ± 3.51^****^	< 0.01	24.05 ± 3.76	23.82 ± 3.53^**^	24.24 ± 3.93^*^	< 0.01
SBP (mmHg)	124.24 ± 13.48	126.46 ± 12.56	123.02 ± 13.83^***^	0.011	128.96 ± 12.76^**^	128.01 ± 12.06	129.74 ± 13.27^**^	< 0.01
DBP (mmHg)	79.08 ± 9.70	81.29 ± 9.39	77.87 ± 9.67^****^	< 0.01	77.09 ± 8.75^**^	77.92 ± 8.72^**^	76.42 ± 8.73^*^, ^***^	0.001
Working status				0.006				< 0.01
Unemployed/housewife/house husband	144 (35.7)	32 (23.4)	112 (42.1)		354 (54.5)	132 (45.2)	222 (62.2)	
Retirement pension	123 (30.5)	22 (16.1)	101 (38.0)		279 (43.0)	147 (50.3)	132 (37.0)	
Laborer	75 (18.6)	48 (35.0)	27 (10.2)		14 (2.2)	12 (4.1)	2 (0.6)	
Technical worker and cadre	61 (15.1)	35 (25.5)	26 (9.8)		2 (0.3)	1 (0.3)	1 (0.3)	
Education level (years)	10.93 ± 3.83	11.94 ± 3.39	10.40 ± 3.95^****^	< 0.01	8.87 ± 4.46^**^	10.00 ± 3.82	7.93 ± 4.74^****^	< 0.01
Cigarette smoking				< 0.01				< 0.01
Never smoker	378 (88.1)	102 (67.5)	276(99.3)		586 (85.9)	212 (69.5)	374 (99.2)	
Former smoker	5 (1.2)	5 (3.3)	0		36 (5.3)	35 (11.5)	1 (0.3)	
Current smoker	46 (10.7)	44 (29.1)	2(0.7)		60 (8.8)	58 (19.0)	2 (0.5)	
Illness history (*n*, %)				0.040				< 0.01
None	136 (31.8)	41 (27.3)	95 (34.2)		88 (12.9)	42 (13.8)	46 (12.2)	
One or two diseases	167 (39.0)	56 (37.3)	111 (39.9)		210 (30.7)	90 (29.5)	120 (31.7)	
Three or more diseases	125 (29.2)	53 (35.3)	72 (25.9)		385 (56.4)	173 (56.7)	212 (56.1)	
Polypharmacy (*n*)				< 0.01				< 0.01
Yes	40 (9.41)	28 (18.79)	12 (4.35)^****^		169 (25.0)^**^	78 (25.9)	91 (24.2)	
HGB (g/L)	133.21 ± 16.15	143.61 ± 16.30	127.52 ± 12.94^****^	< 0.01	129.74 ± 16.07^**^	134.29 ± 17.43^**^	126.04 ± 13.83^****^	< 0.01
FBG (mmol/L)	5.34 ± 1.36	5.60 ± 1.66	5.20 ± 1.15^***^	0.010	5.76 ± 1.73^**^	5.77 ± 1.93	5.75 ± 1.54^**^	< 0.01
SCr (mmol/L)	72.60 ± 18.68	89.52 ± 19.20	63.24 ± 9.62^****^	< 0.01	81.75 ± 26.02^**^	94.71 ± 24.15^*^	71.25 ± 22.53^**^, ^****^	< 0.01
eGFR (mL/min 1.73 m^2^)	90.62 ± 13.36	84.86 ± 14.81	93.80 ± 11.32^****^	< 0.01	74.58 ± 17.18^**^	72.05 ± 16.94^**^	76.62 ± 17.12^**^, ^****^	< 0.01
TG (mmol/L)	1.71 ± 1.49	2.04 ± 1.94	1.54 ± 1.14^****^	0.004	1.42 ± 1.02^**^	1.36 ± 1.25^**^	1.46 ± 0.78	< 0.01
TC (mmol/L)	4.97 ± 1.05	4.91 ± 1.11	5.00 ± 1.02	0.442	4.72 ± 1.23^**^	4.39 ± 1.18^**^	4.99 ± 1.22^****^	0.001
HDL (mmol/L)	1.30 ± 0.40	1.12 ± 0.31	1.39 ± 0.41^****^	< 0.01	1.29 ± 0.35	1.21 ± 0.34^**^	1.35 ± 0.35^****^	0.009
LDL (mmol/L)	2.94 ± 0.87	2.95 ± 0.91	2.94 ± 0.85	0.916	2.81 ± 1.08^*^	2.57 ± 1.00^**^	3.00 ± 1.10^****^	< 0.01
ALT (U/L)	19.27 ± 13.88	22.73 ± 15.69	17.37 ± 12.40^****^	< 0.01	18.11 ± 17.67	19.19 ± 15.93^*^	17.23 ± 18.93	0.026
TBil (µmol/L)	12.65 ± 6.00	13.18 ± 6.50	12.36 ± 5.71	0.180	12.89 ± 7.45	13.86 ± 9.87	12.12 ± 4.55^****^	< 0.01
TP (g/L)	67.67 ± 4.80	66.77 ± 4.71	68.16 ± 4.78^****^	0.004	67.22 ± 5.34	66.27 ± 5.51	68.00 ± 5.07^****^	< 0.01
ALB (g/L)	41.28 ± 3.03	41.09 ± 3.27	41.39 ± 2.88	0.330	39.82 ± 3.21^**^	39.42 ± 3.45^**^	40.14 ± 2.97^**^, ^****^	0.004
ADL	97.86 ± 5.90	96.93 ± 8.63	98.38 ± 3.56^***^	0.049	89.48 ± 14.64^**^	90.03 ± 13.89^**^	89.03 ± 15.22^**^	< 0.01
Frailty scores	0.79 ± 0.91	0.90 ± 1.07	0.72 ± 0.80	0.150	1.41 ± 1.24^**^	1.37 ± 1.15^**^	1.44 ± 1.30^**^	< 0.01
NRS	0.28 ± 0.64	0.35 ± 0.68	0.24 ± 0.62	0.088	1.04 ± 1.12^**^	1.09 ± 1.17^**^	1.00 ± 1.08^**^	< 0.01
MMSE	25.8 ± 3.56	26.51 ± 3.08	25.42 ± 3.74^****^	0.002	21.87 ± 5.90^**^	22.96 ± 5.22^**^	20.98 ± 6.27^**^, ^****^	< 0.01
MoCA	22.73 ± 4.39	23.34 ± 4.44	22.40 ± 4.34^***^	0.038	17.97 ± 5.69^**^	18.83 ± 5.23^**^	17.25 ± 5.96^**^, ^****^	< 0.01
PSQI	8.68 ± 4.22	8.04 ± 3.93	9.02 ± 4.33^***^	0.020	10.00 ± 4.28^**^	9.01 ± 4.09^*^	10.81 ± 4.27^**^, ^****^	< 0.01
HAMD	11.49 ± 4.46	10.51 ± 4.12	12.01 ± 4.55^****^	0.001	13.65 ± 4.88^**^	12.55 ± 4.32^**^	14.55 ± 5.12^**^, ^****^	< 0.01
HAMA	15.26 ± 4.86	14.03 ± 4.76	15.92 ± 4.79^****^	< 0.01	17.18 ± 4.88^**^	15.67 ± 4.39^**^	18.42 ± 4.92^**^, ^****^	< 0.01

Abbreviations: ADL, activities of daily living; ALB, serum albumin; ALT, alanine aminotransferase; BMI, body mass index; DBP, diastolic blood pressure; eGFR, estimated glomerular filtration rate; FBG, fasting blood glucose; HDL‐C, high‐density lipoprotein‐cholesterol; HGB, haemoglobin; LDL‐C, low‐density lipoprotein‐cholesterol; NRS, nutritional risk; SBP, systolic blood pressure; SCr, serum creatinine; TBil, total bilirubin; TC, total cholesterol; TG, triglycerides; T P, total serum protein.

* Significant difference (*p *< 0.05).

** Significant difference (*p *< 0.01) when compared with 60‐year‐old group.

*** Significant difference (*p *< 0.05).

**** Significant difference (*p *< 0.01) when compared with males in the same age group.

### 3.2 Correlation of MMSE and MoCA Scores With Basic Characteristics and Clinical Variables

**FIGURE 1:**
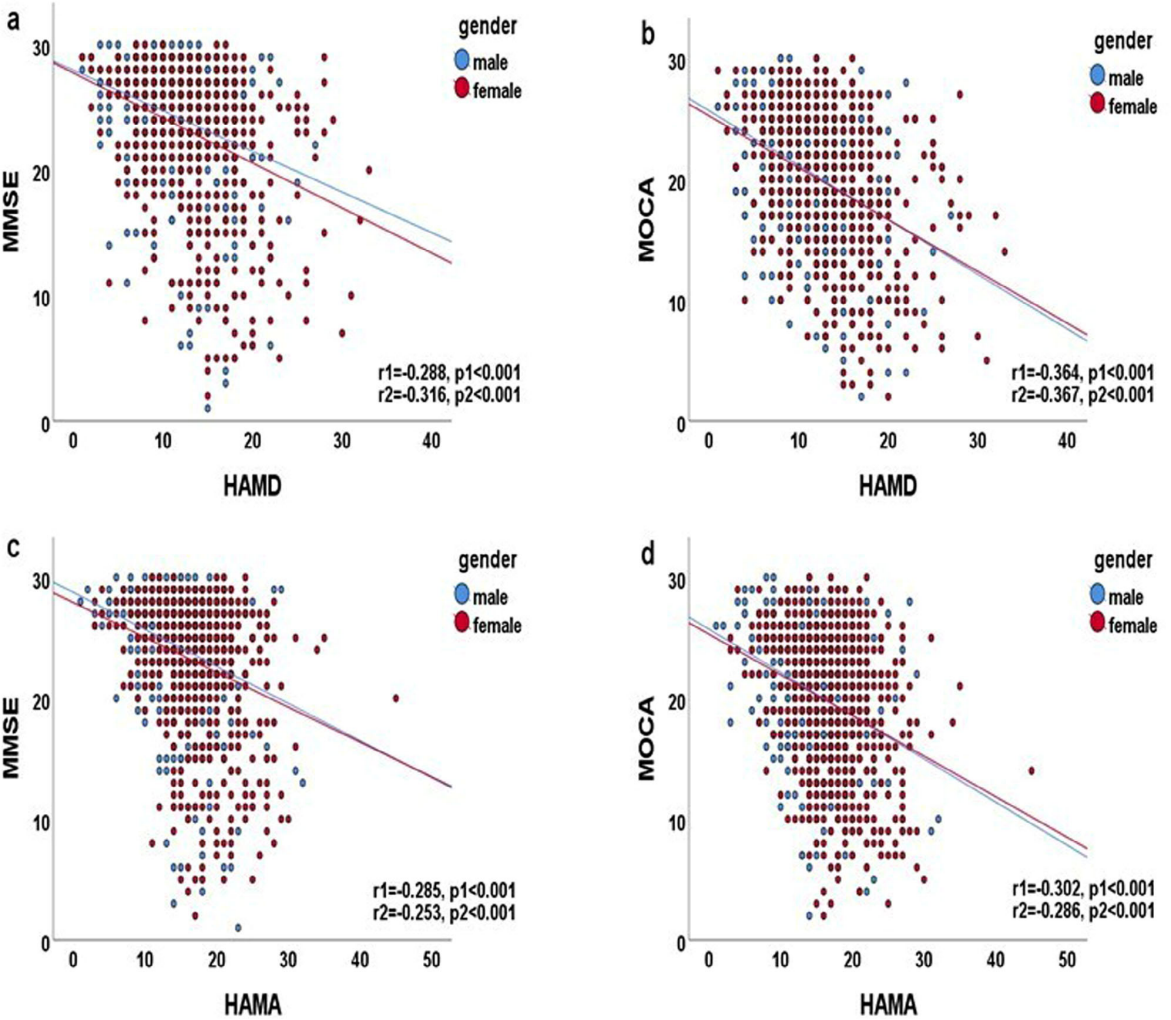
(a) Correlation between MMSE and HAMD (*r* = −0.318, *p* < 0.001). (b) Correlation between MOCA and HAMD (*r* = −0.372, *p* < 0.001). (c) Correlation between MMSE and HAMA (*r* = −0.280, *p* < 0.001). (d) Correlation between MOCA and HAMA (*r* = −0.300, *p* < 0.001). Pearson's correlation analysis was performed; *r* represents the Pearson's correlation coefficient calculated in all participants; *r*1 and *r*2 represent the correlation coefficient calculated in men and women, respectively; *p* represents the *p* value of all participants; *p*1 and *p*2 represent the *p* values of men and women, respectively; blue and red circles indicate men and women, respectively; blue and red straight lines represent the regression lines of men and women, respectively.

**FIGURE 2:**
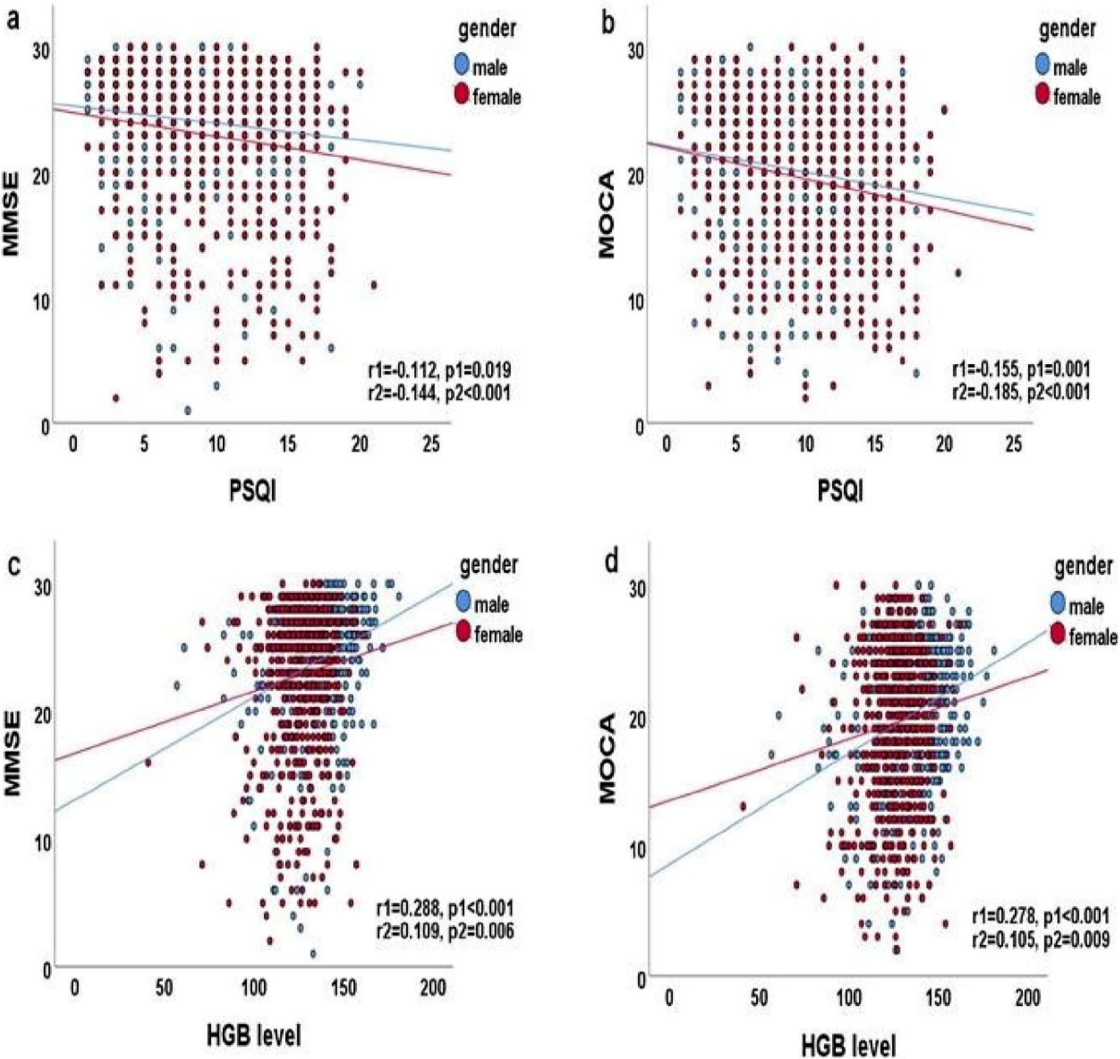
(a) Correlation between MMSE and PSQI (*r* = −0.147, *p* < 0.001). (b) Correlation between MOCA and PSQI (*r* = −0.182, *p* < 0.001). (c) Correlation between MMSE and HGB level (*r* = 0.209, *p* < 0.001). (d) Correlation between MOCA and HGB level (*r* = 0.194, *p* < 0.001). Pearson's correlation analysis was performed; *r* represents the Pearson's correlation coefficient calculated in all participants; *r*1 and *r*2 represent the correlation coefficient calculated in men and women, respectively; *p* represents the *p* value of all participants; *p*1 and *p*2 represent the *p* values of men and women, respectively; blue and red circles indicate men and women, respectively; blue and red straight lines represent the regression lines of men and women, respectively.

Correlations between MMSE and MoCA scores and basic and clinical parameters were established (Tables 2 and 3; Figures 1 and 2). The MMSE and MoCA scores were associated with the HAMD, HAMA, PSQI, NRS‐2002, and Frailty scores and HGB level in all participants (all *p* < 0.001). As shown in Figures 1 and 2, the MMSE and MoCA scores were associated with the HAMD, HAMA, and HGB levels (all *p* < 0.001) and PSQI score (*p* < 0.05) in men. The MMSE and MoCA scores were negatively associated with the HAMD, HAMA, and PSQI (all *p* < 0.001) and positively correlated with the HGB level (*p* < 0.01) in women.

**TABLE 2:** Correlation of MMSE and clinical variables, *Rp* (Spearman's correlation coefficient).

	< 60 years			> 60 years		
	Total	Male	Female	Total	Male	Female
Age (years)	−0.149^**^	−0.097	−0.212^**^	−0.298^**^	−0.264^**^	−0.326^**^
BMI (kg/m^2^)	0.070	0.057	0.018	0.048	0.108	0.007
SBP (mmHg)	−0.072	−0.004	−0.144^*^	−0.089^*^	−0.097	−0.074
DBP (mmHg)	−0.001	0.133	−0.117	0.046	0.031	0.028
Working status	0.414^**^	0.300^**^	0.456^**^	0.421^**^	0.327^**^	0.462^**^
Education level	0.593^**^	0.510^**^	0.605^**^	0.564^**^	0.421^**^	0.639^**^
Cigarette smoking	0.040	−0.077	0.025	0.046	−0.016	−0.061
Illness	−0.099^*^	−0.062	−0.144^*^	−0.002	−0.015	0.002
Polypharmacy	−0.046	−0.033	−0.150^*^	−0.073	−0.066	−0.100
HGB (g/L)	0.093	0.120	0.070	0.198^**^	0.230^**^	0.091
FBG (mmol/L)	0.056	0.100	−0.001	−0.086^*^	−0.090	−0.076
SCr (mmol/L)	0.102^*^	0.023	−0.068	−0.026	−0.094	−0.171^**^
eGFR (mL/min 1.73 m^2^)	0.012	−0.001	0.140^*^	0.193^**^	0.159^**^	0.266^**^
TG (mmol/L)	−0.044	0.007	−0.116	0.020	0.077	0.032
TC (mmol/L)	−0.033	0.033	−0.063	−0.035	0.016	−0.003
HDL (mmol/L)	−0.021	0.017	0.063	−0.053	−0.028	0.000
LDL (mmol/L)	0.002	0.001	0.002	−0.013	0.063	−0.017
ALT (U/L)	0.000	−0.015	−0.056	0.094^*^	0.050	0.098
TBil (µmol/L)	0.042	0.097	−0.013	0.170^**^	0.139^*^	0.174^**^
TP (g/L)	−0.098^*^	0.129	−0.165^**^	−0.180^**^	−0.097	−0.201^**^
ALB (g/L)	−0.017	0.062	−0.053	0.096^*^	0.101	0.119^*^
ADL	0.144^**^	0.149	0.141^*^	0.313^**^	0.341^**^	0.283^**^
NRS	−0.060	−0.196^*^	−0.006	−0.255^**^	−0.206^**^	−0.308^**^
Frailty scores	−0.174^*^	−0.359^**^	−0.078	−0.125^**^	−0.122	−0.137^*^
MoCA	0.737^**^	0.676^**^	0.747^**^	0.862^**^	0.821^**^	0.883^**^
PSQI	−0.177^**^	−0.268^**^	−0.108	−0.095^*^	−0.064	−0.072
HAMD	−0.224^**^	−0.229^**^	−0.176^**^	−0.268^**^	−0.215^**^	−0.277^**^
HAMA	−0.204^**^	−0.274^**^	−0.117	−0.206^**^	−0.216^**^	−0.153^**^

Abbreviations: ADL, activities of daily living; ALB, serum albumin; ALT, alanine aminotransferase; BMI, body mass index; DBP, diastolic blood pressure; eGFR, estimated glomerular filtration rate; FBG, fasting blood glucose; HDL‐C, high‐density lipoprotein‐cholesterol; HGB, haemoglobin; LDL‐C, low‐density lipoprotein‐cholesterol; NRS, nutritional risk; SBP, systolic blood pressure; SCr, serum creatinine; TBil, total bilirubin; TC, total cholesterol; TG, triglycerides; TP, total serum protein.

*Note: p* values are from analysis of variance.

* Significant difference (*p *< 0.05).

** Significant difference (*p *< 0.01).

**TABLE 3:** Correlation of MoCA and clinical variables, *Rp* (Spearman's correlation coefficient).

	< 60 years			> 60 years		
	Total	Male	Female	Total	Male	Female
Age (years)	−0.266^**^	−0.304^**^	−0.269^**^	−0.307^**^	−0.262^**^	−0.352^**^
BMI (kg/m^2^)	0.015	−0.005	−0.030	0.085^*^	0.133^*^	0.051
SBP (mmHg)	−0.111^*^	−0.095	−0.165^**^	−0.106^**^	−0.125^*^	−0.083
DBP (mmHg)	−0.022	0.069	−0.114	0.038	0.047	0.014
Working status	0.511^**^	0.456^**^	0.530^**^	0.479^**^	0.407^**^	0.509^**^
Education level	0.654^**^	0.550^**^	0.693^**^	0.627^**^	0.562^**^	0.658^**^
Cigarette smoking	0.000	−0.098	−0.007	0.029	−0.044	−0.080
Illness	−0.134^**^	−0.153	−0.149^*^	−0.004	−0.050	0.024
Polypharmacy	−0.056	−0.081	−0.115	−0.094^*^	−0.095	−0.113^*^
HGB (g/L)	0.010	−0.053	−0.115	0.209^**^	0.229^**^	0.122^*^
FBG (mmol/L)	0.008	−0.021	−0.007	−0.100^*^	−0.088	−0.094
SCr (mmol/L)	0.131^**^	0.077	0.061	−0.048	−0.135^*^	−0.158^**^
eGFR (mL/min 1.73 m^2^)	−0.035	−0.019	0.042	0.208^**^	0.193^**^	0.261^**^
TG (mmol/L)	−0.047	−0.013	−0.099	0.003	0.066	0.003
TC (mmol/L)	−0.032	0.023	−0.055	−0.052	0.003	−0.034
HDL (mmol/L)	−0.039	−0.084	0.055	−0.063	−0.035	−0.011
LDL (mmol/L)	0.003	0.051	−0.028	−0.020	0.054	−0.032
ALT (U/L)	−0.015	0.007	−0.081	0.080^*^	0.057	0.074
TBil (µmol/L)	0.020	0.055	−0.019	0.183^**^	0.169^**^	0.179^**^
TP (g/L)	−0.113^*^	0.012	−0.153^*^	−0.191^**^	−0.106	−0.212^**^
ALB (g/L)	−0.025	−0.049	−0.020	0.089^*^	0.131^*^	0.091
ADL	0.138^**^	0.105	0.155^*^	0.330^**^	0.338^**^	0.318^**^
NRS	−0.087	−0.143	−0.068	−0.259^**^	−0.209^**^	−0.319^**^
Frailty scores	−0.192^**^	−0.356^**^	−0.096	−0.165^**^	−0.175^*^	−0.172^**^
MMSE	0.737^**^	0.676^**^	0.747^**^	0.862^**^	0.821^**^	0.883^**^
PSQI	−0.200^**^	−0.202^*^	−0.171^**^	−0.121^**^	−0.104	−0.101
HAMD	−0.303^**^	−0.316^**^	−0.263^**^	−0.322^**^	−0.273^**^	−0.335^**^
HAMA	−0.246^**^	−0.327^**^	−0.169^**^	−0.217^**^	−0.200^**^	−0.186^**^

*Note: p* values are from analysis of variance.

Abbreviations: ADL, activities of daily living; ALB, serum albumin; ALT, alanine aminotransferase; BMI, body mass index; DBP, diastolic blood pressure; eGFR, estimated glomerular filtration rate; FBG, fasting blood glucose; HDL‐C, high‐density lipoprotein‐cholesterol; HGB, haemoglobin; LDL‐C, low‐density lipoprotein‐cholesterol; NRS, nutritional risk; SBP, systolic blood pressure; SCr, serum creatinine; TBil, total bilirubin; TC, total cholesterol; TG, triglycerides; TP, total serum protein.

* Significant difference (*p *< 0.05).

** Significant difference (*p *< 0.01).

There was a weak correlation between the MMSE scores and the HAMD and HAMA scores in middle‐aged participants (0.2 < *r* < 0.4; *p* < 0.01). There was a moderate correlation between the MMSE scores and working status and education level (0.4 < *r* < 0.6; *p* < 0.01). The MMSE score was positively correlated with education level (*r* = 0.510; *p* < 0.01) and working status (*r* = 0.300; *p* < 0.01) and negatively correlated with the PSQI, HAMD, HAMA, and Frailty scores (0.2 < *r* < 0.4; *p* < 0.01). The MMSE score was positively correlated with education level (*r* = 0.605; *p* < 0.01) and working status (*r* = 0.456; *p* < 0.01) and negatively associated with age (0.2 < *r* < 0.4; *p* < 0.01) in women.

The MoCA score was correlated with age, the PSQI, HAMD, and HAMA scores (0.2 < *r* < 0.4; *p* < 0.01), working status (*r* = 0.511; *p* < 0.01), and education level (*r* = 0.654; *p* < 0.01) among all middle‐aged participants. The MoCA score was correlated with age, Frailty, the PSQI, HAMD, and HAMA scores (0.2 < *r* < 0.4; *p* < 0.01), working status, and education level (0.4 < *r* < 0.6; *p* < 0.01) in men. The MoCA score was correlated with age, the HAMD score (0.2 < *r* < 0.4; *p* < 0.01), working status (*r* = 0.530; *p* < 0.01), and education level (*r* = 0.693; *p* < 0.01) in females.

The MMSE score was correlated with age, ADL, the NRS‐2002, HAMD, and HAMA scores (0.2 < *r* < 0.4; *p* < 0.01), working status, and education level (0.4 < *r* < 0.6; *p* < 0.01) in the elderly. The MMSE score was correlated with age, working status, HGB, the ADL, NRS‐2002, HAMD, and HAMA scores (0.2 < *r* < 0.4; *p* < 0.01), and education level (*r* = 0.421; *p* < 0.01) in men. The MMSE score was correlated with age, the estimated glomerular filtration rate (eGFR), the TP level, the ADL, NRS‐2002, and HAMD scores (0.2 < *r* < 0.4; *p* < 0.01), working status (*r* = 0.462; *p* < 0.01), and education level (*r* = 0.639; *p* < 0.01) in women.

The MoCA score was correlated with age, the HGB level, the eGFR, the ADL, NRS‐2002, HAMD, and HAMA scores (0.2 < *r* < 0.4; *p* < 0.01), working status (*r* = 0.479; *p* < 0.01), and education level (*r* = 0.627; *p* < 0.01) among elderly participants. The MoCA score was correlated with age, the HGB level, the ADL, NRS‐2002, HAMD, and HAMA scores (0.2 < *r *< 0.4; *p* < 0.01), working status, and education level (0.4 < *r* < 0.6; *p* < 0.01) in men. The MoCA score was correlated with age, the eGFR, the TP level, the ADL, NRS‐2002, and HAMD scores (0.2 < *r* < 0.4; *p* < 0.01), working status (*r* = 0.509; *p* < 0.01), and education level (*r* = 0.658; *p* < 0.01) in women.

### 3.3 Relationship Between the MMSE and MoCA Scores and Clinical Variables Using a Stepwise Multiple Regression Model

The clinical parameters independently associated with the MMSE and MoCA scores were examined using a multiple regression model with stepwise entry (Table 4, Bartrés‐Faz et al. 2023). Independent predictors of cognitive impairment included in the model were all parameters that were correlated with the MMSE and MoCA scores in the pairwise correlation analysis and traditional cognitive risk factors. After full adjustment, the MMSE score had a significant and independent association with age, education level, HGB level, and the ADL, PSQI, and HAMD scores (*p* < 0.05). The MoCA score was significantly associated with age, education level, working status, HGB and FBG levels, and the ADL, PSQI, and HAMD scores (*p* < 0.05).

**TABLE 4:** Relationship between MMSE and MoCA and clinical variables using a stepwise multiple regression model.

	MMSE				MOCA			
	Model 1		Model 2		Model 1		Model 2	
	Beta	*p*	Beta	*p*	Beta	*p*	Beta	*p*
Age (years)	−0.136^**^	0.001	−0.166^**^	0.000	−0.180^**^	0.000	−0.223^**^	0.000
Working status	0.023	0.511	0.049	0.196	0.093^**^	0.003	0.098^**^	0.002
Education level	0.518^**^	0.000	0.542^**^	0.000	0.523^**^	0.000	0.518^**^	0.000
HGB (g/L)	0.076^*^	0.019	0.068^*^	0.022	0.079^**^	0.007	0.057^*^	0.035
FBG (mmol/L)	−0.050	0.100	−0.053	0.065	−0.072^**^	0.009	−0.079^**^	0.003
ADL	0.177^**^	0.000	0.188^**^	0.000	0.130^**^	0.000	0.134^**^	0.000
PSQI	0.076^*^	0.037	0.086^*^	0.016	0.063	0.057	0.077^*^	0.018
HAMD	−0.120^*^	0.010	−0.137^**^	0.000	−0.171^**^	0.000	−0.158^**^	0.000

*Note*: Standardized coefficients and *p* values were the outcome of regression analyses.

Abbreviations: Beta, standardized coefficients; Model 1, unadjusted model; Model 2, fully adjusted for age, gender, BMI, SBP, DBP, HGB, FBG, TG, TC, LDL, SCr, eGFR, ALT, TBil, TP, ALB, working status, education level, illness, ADL, frailty scores, NRS, PSQI, HAMD, and HAMA.

* Significant difference (*p *< 0.05).

** Significant difference (*p *< 0.01).

## 4 Discussion

In the current study a sex‐ and age‐specific analysis was performed between cognitive function, mood, sleep, frailty, and nutrition in geriatrics patients. In addition, an extensive list of associated factors was generated. Research focusing on risk factors for cognitive impairment among elderly Chinese has been limited. Some gaps remain to be filled, including differences in cognitive function change by age, sex, illness status, and psychological conditions. The purpose of this study was to analyze risk factors for cognitive impairment to establish medical interventions or strategies to delay cognitive decline.

The main findings of the current study were as follows: (1) There were sex and age differences in cognition, mood, sleep quality, and some sociodemographic features, such as education level and working status. The elderly had significantly lower levels of education, working status, MMSE and MoCA scores and higher levels of PSQI, HAMD, HAMA, NRS‐2002, and Frailty scores. Women and elderly were more susceptible to affective disorders, sleep disturbances, and cognitive impairment. (2) Age, education level, working status, HGB level, and ADL, HAMD, and PSQI scores were significantly and independently associated with cognitive impairment after full adjustment, especially in elderly women. (3) The association between the serum HGB level and cognitive function was confirmed. Women and elderly had significantly lower levels of HGB. (4) Some risk factors, such as frailty and nutritional risk, were significantly associated with cognitive impairment, even in middle age, suggesting that interventions should be implemented before age‐related accelerated cognitive changes or early stages of dementia. These findings may contribute to develop multidomain intervention measures for decreasing dementia risk. The results also highlighted the psychosocial support needs among the elderly and women, who were more emotionally distressed and reported higher cognitive impairments and sleep disturbances.

Women had a higher risk of cognitive impairment developing than men in the current study, which was in agreement with previous research findings (Manly et al. 2005; Petersen et al. 2010). However, some studies in other countries (Italy, Germany, and Finland) did not detect any differences based on sex (Manly et al. 2005). In China, the increased prevalence of cognitive impairment in women may be because women had less access to education than men in the past (Xue et al. 2018). In the current study, it was confirmed that the education level and the working status of women were lower than men, especially among elderly females. However, this phenomenon gradually improved (Zhang et al. 2019). Young people have higher levels of education, and there was no sex difference in their working status. More research is clearly needed to explain the relationship between sex and the prevalence of cognitive impairment.

Our findings were in line with the results of previous studies that observed cognitive impairment was highly related to depression and anxiety (Han et al. 2021; Cladder‐Micus et al. 2018; Götze et al. 2020). In addition, important demographic factors and clinical variables were analyzed. The MMSE and MoCA scores were significantly and independently associated with the HAMD score (all *p* < 0.01), especially in elderly females. Thus, our results demonstrated that depression was an independent risk factor for cognitive impairment. Compared to men, women are prone to experience affective disorders, such as anxiety and depression, with higher fatigue and sleep disturbances than men, especially in elderly women, although the sex gap is smaller than young (Niu et al. 2023). Factors that have been proposed to explain these findings include hormonal differences, different sex roles, social and cultural norms, disadvantages and empowerment throughout one's life, and the coping styles of elderly men (Faravelli et al. 2013). However, little research has fully proved these explanations. Understanding the impact of sex on cognitive impairment in the elderly has potential benefits if a broader understanding is systematically measured, which need to be verified with larger studies.

In this study significant differences in PSQI scores were detected between the age and sex groups. Women and the elderly were more likely to have sleep disorders, which was consistent with previous findings (Ma et al. 2020; Xu et al. 2022). The MMSE and MoCA scores remained significantly associated with the PSQI score after controlling for confounding factors. Previously, a strong association was confirmed between cognitive function and sleep characteristics (Hu et al. 2021). Considering the inter‐relationship between sleep disturbances, cognitive impairment, and depression among the elderly, important confounding factors, such as anxiety and depression, were further analyzed, the results of which improved previous research. Our results demonstrated that sleep quality had a significant and independent relationship with cognitive function after full adjustment. The mechanisms behind the relationship between sleep and cognition are intricately complex and may be related to neurotransmitters and cerebral metabolism (Falck et al. 2018; Moraes et al. 2006; Mizuno et al. 2004; Taber and Hurley 2006). Although its true function is currently unclear, sleep is crucial for maintaining brain health throughout older adulthood and critical to cognitive function (Sewell et al. 2021). Further research is needed to understand the mechanisms underlying the relationship between sleep, emotion, and cognition. Nevertheless, the current study observed that sleep and emotion influence cognitive function through shared and independent pathways. Therefore, improving sleep and emotion may prevent the development of cognitive deficits.

The relationship between the serum HGB level and cognitive function has been analyzed in previous studies. Most previous studies conclude that anemia has a negative correlation with cognition (Marzban et al. 2021; Wang et al. 2023), which agrees with our study. Sex differences in the HGB levels existed in women in the current study (*p* < 0.01). Women and the elderly have significantly lower levels of HGB. After full adjustment, the MMSE and MoCA scores remained significantly associated with the HGB level. The mechanism underlying the HGB level and cognitive impairment may be connected to cerebrovascular factors, oxidative stress in the brain, interference with metabolism, frailty, and nutrition effects (Yang et al. 2022; Shah et al. 2009). However, why female cognitive function is more significantly affected by the HGB level is unclear and further research is warranted.

In the current study we also observed an increased Frailty score, nutrition risk, and limited ADL, which were significantly associated with cognitive impairment and agreed with previous studies (Robinson et al. 2022; Dent et al. 2019; Leist et al. 2020; Wirth et al. 2011; Chen et al. 2022). However, after full adjustment, only the independent correlation between ADL scores and cognitive impairment persisted. Another interesting aspect of this study was that the findings were observed among middle‐aged individuals, suggesting that some risk factors for cognitive impairment could already be identified and intervened before age‐related accelerated cognitive changes or the earliest stage possible. In addition, our study confirmed that the MoCA was more sensitive than the MMSE in detecting and analyzing mild cognitive impairment in middle‐aged and elderly people and might be more useful as a test for general cognitive screening aims (Guo et al. 2016; Jia et al. 2021).

The strength of the current study included an analysis on cognitive function and multiple risk factors from a comprehensive perspective according to age and sex by considering various factors, such as sociodemographics, lifestyle, medical history, psychological factors, and clinical variables. However, some limitations need to be emphasized. First, this was a cross‐sectional study, with participants only enrolled from our hospital. Thus, the general implications of the data were limited. A longitudinally designed study, with participants from different regions of China, is required to confirm these findings. Second, due to the limitation in terms of measurement method and variable selection, it is necessary to conduct research by evaluating more important metrics and variables in the future, including specific cognitive domains and various aspects of confounding factors. Third, the cross‐sectional design and study methods appear to be designed more for identifying predictors rather than establishing causality. More carefully and advanced epidemiological and statistical methods should be designed in the future research.

## 5 Conclusion

This study examined the association between cognition, sleep, emotion, and other aspects of elderly patients. Our results demonstrated that age, education level, working status, HGB level, and ADL, HAMD, and PSQI scores were significantly and independently associated with cognitive impairment. We found that elderly and female patients were prone to cognitive impairment, anemia, sleep disorders, anxiety, and depression. Our results emphasized the importance of early detection and prevention to slow progression to cognitive impairment. Support services should be improved among the elderly and women, who are more emotionally distressed and have cognitive impairments and sleep disturbances. By improving sleep, relaxing emotions, preventing anemia, and adjusting lifestyle, cognitive deficits can be reduced. Further investigation is required to confirm and improve these findings.

## Author Contributions

**Nan Wang**: writing–original draft, writing–review and editing, methodology, data curation, investigation, formal analysis, conceptualization. **Qunying Zhang**: investigation, validation, supervision, funding acquisition, project administration; writing–review and editing, visualization. **Peng Li**: investigation, formal analysis, resources, visualization, software, validation. **Lilan Guo**: data curation, methodology. **Xiaoman Wu**: data curation, methodology, software. **Qiuyun Tu**: conceptualization, investigation, funding acquisition, writing–review and editing, methodology, data curation, supervision, resources, project administration.

## Conflicts of Interest

The authors declare no conflicts of interest.

### Peer Review

The peer review history for this article is available at https://publons.com/publon/10.1002/brb3.70170.

## Data Availability

The data that support the findings of this study are available from the corresponding author upon reasonable request.
